# The Era of Immunotherapy in Hepatocellular Carcinoma: The New Mission and Challenges of Magnetic Resonance Imaging

**DOI:** 10.3390/cancers15194677

**Published:** 2023-09-22

**Authors:** Yidi Chen, Chongtu Yang, Liuji Sheng, Hanyu Jiang, Bin Song

**Affiliations:** 1 Department of Radiology, West China Hospital, Sichuan University, Chengdu 610064, China; chenyidi1152@126.com (Y.C.); henrys1011@163.com (C.Y.); shenglj_1999@163.com (L.S.); 2 Department of Radiology, Sanya People’s Hospital, Sanya 572000, China

**Keywords:** hepatocellular carcinoma, immunotherapy, magnetic resonance imaging, artificial intelligence

## Abstract

**Simple Summary:**

Clinicians should be aware that the immune microenvironment not only influences biological characteristics of HCC but also provides a reliable theoretical basis for comprehensively evaluating tumor responses after immunotherapy. Combining multi-parameter MRI with radiomics and deep learning can help to explore the biological characteristics of HCC. Future artificial intelligence technologies may extract biologically interpretable multidimensional structures from MRI images more effectively, thereby promoting precise and visual classification of HCC immune microenvironments. This advancement of MRI combined with multi-omics could potentially assist in individualized treatment planning.

**Abstract:**

In recent years, significant advancements in immunotherapy for hepatocellular carcinoma (HCC) have shown the potential to further improve the prognosis of patients with advanced HCC. However, in clinical practice, there is still a lack of effective biomarkers for identifying the patient who would benefit from immunotherapy and predicting the tumor response to immunotherapy. The immune microenvironment of HCC plays a crucial role in tumor development and drug responses. However, due to the complexity of immune microenvironment, currently, no single pathological or molecular biomarker can effectively predict tumor responses to immunotherapy. Magnetic resonance imaging (MRI) images provide rich biological information; existing studies suggest the feasibility of using MRI to assess the immune microenvironment of HCC and predict tumor responses to immunotherapy. Nevertheless, there are limitations, such as the suboptimal performance of conventional MRI sequences, incomplete feature extraction in previous deep learning methods, and limited interpretability. Further study needs to combine qualitative features, quantitative parameters, multi-omics characteristics related to the HCC immune microenvironment, and various deep learning techniques in multi-center research cohorts. Subsequently, efforts should also be undertaken to construct and validate a visual predictive tool of tumor response, and assess its predictive value for patient survival benefits. Additionally, future research endeavors must aim to provide an accurate, efficient, non-invasive, and highly interpretable method for predicting the effectiveness of immune therapy.

## 1. Introduction

Primary liver cancer (PLC) is the fourth most common malignant tumor worldwide and the second leading cause of cancer-related deaths. PLC poses a significant threat to people’s lives and health. Approximately 75% of PLC cases are attributed as hepatocellular carcinoma (HCC) [1]. Due to its insidious onset, less than 30% of patients are eligible for curative treatment upon initial diagnosis [2]. Immunotherapy plays a significant role in the management of advanced HCC, aiding in disease control and prolonging patient survival [3].

The development and progression of HCC involve a multifactorial and multistep process, including interactions between hepatitis B virus, hepatitis C virus infection, and chronic liver inflammation response [4]. Increasing evidence reveals that immune mechanisms play a critical role in the occurrence and progression of HCC. Targeting the immune microenvironment system of HCC has the potential to improve the prognosis of HCC patients [5].

The PD-1/PD-L1 and CTLA-4 immune checkpoint pathways within the immune microenvironment of HCC are among the important mechanisms by which tumors induce immune escape [6]. This mechanism provides a theoretical basis for the use of PD-1 or PD-L1 immune checkpoint inhibitors. In recent years, emerging immunotherapeutic approaches such as immune checkpoint inhibitors (ICIs) have made significant advancements in the treatment of HCC and have become an integral part of systemic anti-tumor therapy for advanced-stage HCC. Combination therapy with atezolizumab and bevacizumab demonstrated superiority over sorafenib in the phase III IMBRAVE-150 study, leading to its FDA approval as a first-line treatment for advanced HCC [7].

Subsequently, although a series of randomized, open-label phase II/III clinical studies have reported favorable overall survival and progression-free survival benefits of immune checkpoint inhibitors (ICIs) in patients with advanced HCC, the objective response rate of the lesions is still at a low level (approximately 25%) [8,9]. Studies have shown that around 70–80% of patients experience treatment failure with immunotherapy due to immune microenvironment status such as “immunological desert” or “immune exclusion” within the tumor [10,11], revealing that the different states of the tumor immune microenvironment directly affect the therapeutic response to ICIs. A randomized controlled phase I/II clinical study investigating the combination of tremelimumab and durvalumab in unresectable HCC patients demonstrated a correlation between early CD8+ T cell expansion and tumor response in each group [12], further emphasizing the significant impact of the immune microenvironment on immunotherapy.

The current expert consensus suggests that due to the relatively short clinical application time of immunotherapy for HCC, there is still a lack of experience in various aspects, including patient selection, treatment regimen determination, and efficacy assessment [13]. Furthermore, different patients exhibit varying responses to immunotherapy, and some patients may experience severe immune-related adverse events following treatment [14]. Therefore, predicting tumor response after HCC immunotherapy through assessing the tumor immune microenvironment status holds significant values.

## 2. The Potential Clinical Significance of Tumor Immune Microenvironment Classification in Hepatocellular Carcinoma

To gain a deeper understanding of the potential impact of different states of the tumor immune microenvironment on tumor development and the response to tumor immunotherapyin hepatocellular carcinoma (HCC), some researchers have classified the tumor immune microenvironment based on relevant information about the enrichment of major immune cells. Kurebayashi et al. [15] classified the immune microenvironment of HCC into three immunosubtypes: immune-high, immune-mid, and immune-low. They revealed that these subtypes have an additional prognostic impact on the histological and molecular classification of HCC. Sia et al. [16] validated the “immune subtype HCC “using transcriptomics and other methods, showing that the “immune subtype HCC” characterized by high immune infiltration and increased PD-1/PD-L1 signaling pathway, leading them to exhibit a better response to ICIs. Llovet et al. [17] reported four distinct immune subtypes of HCC: immune active, immune exhausted, immune intermediate, and immune excluded. Among these subtypes, immune active and immune exhausted are considered as immune-related tumors characterized by distinct immune cell infiltrates, and they typically exhibit responsiveness to ICIs. Videlicet or immune active tumors are characterized by the abundant infiltration of active helper T cells (CD4+) and cytotoxic T cells (CD8+), as well as enrichment of M1-type macrophages. Immune exhausted tumors are predominantly driven by TGF-β-induced CD8+ T cell exhaustion. Intermediate tumors are enriched with TP53 mutations, chromosomal instability, and frequent loss of interferon signaling or antigen presentation-related genes. On the other hand, immune excluded tumors are characterized by a lack of CD8+ T cells and an increase in Treg cells, resulting in an “immunologically cold” environment driven by overexpression of the Wnt-β-catenin signaling pathway and other immune checkpoint cascades. Immune intermediate and immune excluded tumors are considered as immune-suppressive tumors, and they exhibit resistance to ICIs [18].

In addition, the 2020 European Association for the Study of the Liver (EASL 2020) proposed a new immune subtype based on the molecular characteristics of HCC, known as the “Immune-like” subtype. This novel subtype shares similarities with the immune active tumors, exhibiting strong interferon signaling activation and immune activity. However, it also presents CTNNB1 mutations. Specifically, interferon-gamma (IFN-γ) and its induced production of CXCL9 and CXCL10 contribute to the establishment of an inflammatory tumor microenvironment, which plays a role in the tumor’s response to ICIs therapy.

**Figure 1:**
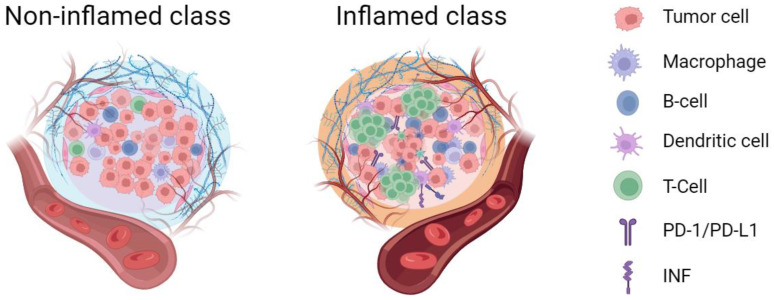
The inflamed and non-inflamed class of tumor immune microenvironment. The inflamed tumor immune microenvironment exhibited a higher degree of immune cell infiltration compared to the non-inflamed microenvironment (figure created with BioRender.com (accessed on 25 August 2023)).

Based on the aforementioned studies, an important review article published by Llovet et al. in 2022 [3] provides a detailed definition and description of the immune subtypes of HCC. They categorized HCC into the inflamed class and non-inflamed class based on the cellular and molecular characteristics (Figure 1). The inflamed class includes the immune active, exhausted, and immune-like subtypes, while the non-inflamed class includes the intermediate and excluded subtypes. The inflamed class accounts for approximately 30–35% of HCC tumors; recent studies have confirmed that it is the most common subtype among HCC patients who respond to anti-PD-1/PD-L1 therapy [19]. Furthermore, around 65–70% of HCC tumors belong to the non-inflamed class, which shows poor response to ICIs and is typically not suitable for standalone immunotherapy. Instead, treatment approaches such as anti-angiogenic agents, tyrosine kinase inhibitors, or TACE are recommended more for non-inflamed HCC. The introduction of immune microenvironment subtyping partially addresses the lack of reliable biomarkers for predicting tumor response to HCC immunotherapy. Additionally, the literature has reported that VEGF pathway inhibitors can synergize with ICIs, potentially stimulating immune cell infiltration in HCC treatment. This conversion of non-inflamed “cold tumors” into inflamed “hot tumors” enhances the efficacy of ICIs [3]. Therefore, monitoring the dynamic changes in the tumor immune microenvironment status during treatment also holds important clinical significance.

## 3. The Challenges of Accurately Subtyping the Tumor Immune Microenvironment in Hepatocellular Carcinoma

Increasing evidence suggests that achieving accurate subtyping of the tumor immune microenvironment in HCC requires attention to the spatial heterogeneity of the immune microenvironment. This spatial heterogeneity not only affects the accuracy of tumor immune microenvironment subtyping but also provides important clues for treatment decision-making [20].

Research has found that small tumors in patients with multifocal HCC exhibit higher immune cell infiltration and immune pathway upregulation compared to larger tumors. This finding partially explains why small and large tumors within the same case may have different responses to anti-PD-1 therapy [21]. Lancet Oncology reported that PD-L1 expression in different regions of immune cells and tumor cells varies, and the combined positive score is associated with the treatment response of pembrolizumab [22]. Zheng et al. [23] found an enrichment of CD4/CD8/PD-1 triple-positive T cells at the tumor front, which indicates a better prognosis. Deep single-cell sequencing results showed higher expression of exhausted CD8+ T cells and Treg cells in HCC tissue compared to peri-tumoral liver tissue [24]. Additionally, a team from the National Cancer Center of China used spatial transcriptomics to describe the distribution of immune cells in HCC tumors and their interactions with tumor clusters, revealing spatial heterogeneity in the immune microenvironment of HCC. Further research on these heterogeneities has the potential to improve personalized treatment and drug development [25].

Currently, the methods available for partially achieving phenotypic discrimination and spatial analysis of the HCC immune microenvironment include (I) in situ hybridization techniques, including fluorescence in situ hybridization (FISH) and multiplexed error-robust fluorescence in situ hybridization (MERFISH). The former enables qualitative or quantitative analysis of DNA, providing some rudimentary spatial information. The latter allows for highly multiplexed transcriptomic analysis at the single-cell level with spatial resolution. However, these methods can detect only a limited number of known target genes simultaneously [26]. (II) Spatial transcriptomics techniques have overcome the limitations of in situ hybridization. By using unique positional barcodes to locate frozen tissue sections on an array of reverse transcription primers, this approach provides high-quality whole-genome transcriptomic data and complete two-dimensional spatial information. However, the main limitation of this method is its relatively low spatial resolution [27]. (III) Imaging Mass Cytometry (IMC) technology enables the phenotypic characterization of the tumor microenvironment based on spatial resolution. IMC utilizes the binding of antibodies to heavy metals for staining individual tissue sections. By employing a high-resolution laser ablation system, the sample is ablated point-by-point, allowing for extensive cellular analysis of the ablated material. Thus, while preserving cellular spatial information, IMC can simultaneously detect dozens of proteins and protein modifications, enabling the determination of a spatially resolved single-cell phenotype. As a result, IMC has become a practical tool for the topological analysis of the tumor microenvironment [28].

Above all, the classification of HCC tumor immune microenvironment helps to better characterize the distinct features of the immune microenvironment, thereby enabling more accurate prediction of tumor immunotherapy response. However, the current examination methods for immune cells, molecular phenotypes and spatial distribution have certain limitations, such as the following: (I) High technical requirements, limiting their implementation to specialized laboratories and making routine application in hospitals challenging. (II) High demands for tissue sample preservation and relatively complex processing steps during examination. (III) Limited coverage of the examined area, resulting in the loss of some in situ tissue structure and adjacent area information. (IV) Difficulty in obtaining gross pathological specimens from HCC patients receiving immune-related treatments, as results from needle biopsies can be influenced and may not fully reflect the spatial heterogeneity of the tumor. (V) Inability to achieve real-time dynamic monitoring of the tumor immune microenvironment in HCC patients.

Therefore, there is an urgent need for an accurate, efficient, and convenient detection method to systematically and comprehensively evaluate the immune microenvironment phenotypes of individual tumors and their surrounding tissues. Especially, a useful tool is needed in clinical practice that would enable non-invasive prediction of the efficacy of immunotherapy and real-time dynamic monitoring of changes in the tumor immune microenvironment during treatment.

## 4. The Current Status of Non-Invasive Evaluation of Tumor Immune Microenvironment Using Magnetic Resonance Imaging

**Figure 2:**
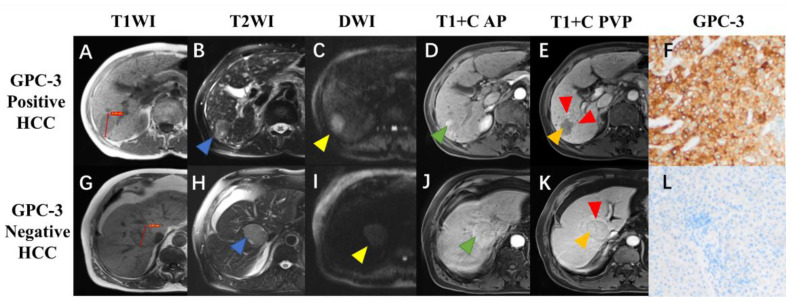
Gadolinium ethoxybenzyl-diethylenetriaminepentaacetic acid (Gd-EOB-DTPA)-enhanced MRI and histopathologic images of hepatocellular carcinoma (HCC) with different glypican-3 (GPC-3) expressions. A 72-year-old male patient with GPC-3-positive expression HCC (**A**–**F**), and a 77-year-old male patient with GPC-3-negative expression HCC (**G**–**L**). Pre-contrast T1-weighted images showed a hypointense lesion (3.54 cm) in the right liver (**A**) and a hypointense lesion (3.97 cm) in the mid liver (**G**); T2-weighted images showed hyperintense lesions with ((**B**), blue arrow) and without ((**H**), blue arrow) “iron sparing in solid mass”; diffusion-weighted images demonstrated the presence ((**C**), yellow arrow) and absence ((**I**), yellow arrow) of “marked diffusion restriction”; arterial phase images showed “nonperipheral-nonglobal arterial phase hyperenhancement” ((**D**), green arrow) and “global arterial phase hyperenhancement” ((**J**), green arrow); portal venous phase images showed “nonperipheral washout” and infiltrative appearance ((**E**), orange and red arrow) and “no-washout” and smooth margin ((**K**), orange and red arrow); immunohistochemical staining revealed the GPC-3-positive (**F ×100**) and negative (**L ×100**) expressions (Reprinted/adapted with permission from Ref. [30]. Copyright 2022, copyright owner Bin song).

In recent years, numerous researchers have made significant progress in the non-invasive evaluation of the immune microenvironment using magnetic resonance imaging (MRI), which has gradually become a research hotspot in this field. For instance, GPC-3, besides being a diagnostic biomarker for HCC, also plays a crucial role in the tumor immune microenvironment. It is considered as a potential target for the next generation of immune therapies [29]. Chen et al. demonstrated a predictive model based on serum AFP levels and Gadolinium ethoxybenzyl-diethylenetriaminepentaacetic acid (Gd-EOB-DTPA)-enhanced MRI features, which accurately assesses GPC-3 expression in HCC tumor tissue (Figure 2) [30]. Due to Gd-EOB-DTPA-enhanced MRI demonstrating OATP1B1/B3-dependent hepatocyte-specific uptake, the expression of OATP1B1/B3 being associated with the WNT-β-catenin signaling pathway [31], and the immune cell infiltration of the tumor microenvironment [32], it can be potentially used for evaluating the immune microenvironment status of HCC.

In a study conducted by Professor Wang [33], Gd-EOB-DTPA-enhanced MRI was used to predict CD8 cell density and PD-L1 expression in HCC tumors. The results showed that MRI features such as irregular tumor margin, low signal intensity in the hepatobiliary phase, and lack of capsule could potentially identify HCC patients in an immune-activated state and predict the outcomes of immunotherapy. Another study [34] indicated that MRI characteristics based on tumor number, intratumoral vessels, margin morphology, capsule formation, marginal enhancement, and T1 relaxation time could effectively predict the immune scores of HCC tumors. A novel research conducted at the Imaging Institute of Vanderbilt University utilized the MRI-restricted diffusion sequence (IMPULSED), which can depict intracellular structures and variations in cell size, to assess T-cell infiltration in tumor tissue [28,35]. The results demonstrated that IMPULSED accurately reflects the extent of T-cell infiltration, suggesting its potential value as an imaging biomarker for evaluating the effectiveness of ICIs therapy.

Positron emission tomography (PET) imaging has been proven to be a valuable noninvasive tool for evaluating the tumor microenvironment of HCC. For instance, Zhou et al. [36] reported that GPC3-targeted PET imaging not only improves early HCC detection- but also effectively predicts the prognosis of anti-GPC3 cytotoxic antibody immunotoxin therapies. Itoh et al. [37] demonstrated that a high SUV_max_ on fluorine-18 fluorodeoxyglucose PET-computed tomography (PET/CT) is associated with a poor clinical outcome and PD-L1 expression in patients with HCC. Furthermore, a one-armed antibody positron emission tomography tracer, 89ZED88082A, can visualize immunohistochemically confirmed CD8+ T cells in solid tumors by showing the tissue radioactivity localized areas [38].

In addition to conventional imaging features for assessing the tumor immune microenvironment, radiomics has emerged as a viable approach in recent years for tumor diagnosis, microenvironment evaluation, and prognostic assessment [39]. Radiomics utilizes automated computer algorithms to perform quantitative analysis of images, objectively quantifying the heterogeneity of images by measuring spatial variations in pixel intensity [40]. It can quantify tumor heterogeneity associated with microenvironmental changes, including cellular architecture, extracellular matrix deposition, angiogenesis, necrosis, and fibrosis [41]. Gong et al. [42] revealed that a radiomics model based on multisequence MRI has the potential to predict the preoperative expression of PD-1 and PD-L1 in HCC. Both Song et al. [43] and Roger et al. [44] have validated that radiomic features can predict the infiltration of CD8+ T cells in HCC tumor tissue. Tian et al. [45] confirmed that radiomic features based on EOB-DTPA-enhanced MRI can predict the density of CD3+ and CD8+ T cells in HCC. Furthermore, Stefanie et al. [46] found a high correlation between radiomic features of MRI and the expression of CD3, CD68, and CD31, which can reflect the levels of PD-L1 at the protein level, as well as the expression levels of PD-1 and CTLA-4 at the mRNA level. Additionally, the present study indicates that utilizing MRI radiomic features can enable noninvasive prediction of PD-L2 expression in HCC prior to surgery. This information can serve as a valuable reference for guiding the selection of ICIs therapy [47].

## 5. Can MRI Accurately Predict the Efficacy of Immunotherapy in Hepatocellular Carcinoma before Treatment?

In the era of immunotherapy for HCC, the biggest challenge in clinical practice is the inability to predict which patients may have the greatest survival benefit from immunotherapy and which patients do not respond to immunotherapy. If we could accurately predict patients who are completely resistant to immunotherapy, it would not only save expensive medical resources, but more important, prevent these patients from receiving unnecessary immunotherapy drugs, thereby avoiding potential risks associated with drug side effects. Due to the immense tumor heterogeneity in HCC, tissue specimens obtained through pre-treatment biopsies are often unable to fully and accurately reflect the overall immune microenvironment of the tumor. Consequently, there is no current reliable biomarker in clinical practice to predict the efficacy of immunotherapy in HCC.

As mentioned above, multi-parametric MR imaging contains a wealth of biological features, enabling non-invasive, comprehensive, and dynamic assessment of the immune microenvironment status in HCC (Table 1). Kudo M. indicated that Gd-EOB-DTPA-enhanced MRI could predict wnt/β-catenin mutation and resistance to immune checkpoint inhibitor therapy in HCC [48]. Similarly, Sasaki et al. [49] proposed that the hepatobiliary phase (HBP) of Gd-EOB-DTPA-enhanced MRI was useful for predicting the therapeutic effect of atezolizumab plus bevacizumab therapy on unresectable HCC. Aoki et al. [50] also illustrated higher enhancement intrahepatic nodules on the HBP of Gd-EOB-DTPA-enhanced MRI as a poor responsive marker of Anti-PD-1/PD-L1 monotherapy for unresectable hepatocellular carcinoma. Additionally, radiomics as a powerful analytical method for quantifying these microscopic features provide a feasible and non-invasive approach for monitoring the ICI response, showing a new promising direction [51]. In addition to Gd-EOB-DTPA-enhanced MRI, intravoxel incoherent motion (IVIM) has also proven effective in predicting tumor response to Atezo + Bev therapy. The presented research reveals that changes in the pre- and post-treatment true diffusion coefficients on MRI, which may reflect alterations in the tumor immune microenvironment following treatment, are significantly associated with improved progression-free survival [52].

Presented studies have indicated that 20–30% of HCC patients categorized under the immune exclusion class may exhibit resistance to ICIs therapy. This resistance is attributed to diminished infiltration of CD8+ T-cells into tumors, caused by mutations in the WNT/β-catenin pathway and the absence of PD-L1 expression in cancer cells [53,54]. Evidence demonstrated Gd-EOB-DTPA-enhanced MRI can be utilized to predict the occurrence of the WNT/β-catenin mutation, which activates OATP1B3 [31]. This suggests that Gd-EOB-DTPA-enhanced MRI has potential clinical utility in identifying the HCC classification of immune exclusion, which is unlikely to respond favorably to ICIs monotherapy [55]. ICIs monotherapy was ineffective and even led to significant disease progression in HCC cases with WNT/β-catenin mutation, as indicated by a high-intensity signal in the HBP of Gd-EOB-DTPA-enhanced MRI. Moreover, HCC patients with a high-intensity signal in the HBP of Gd-EOB-DTPA-enhanced MRI who received ICI monotherapy may experience poorer progression-free survival compared to those with a low-intensity signal in the HBP of Gd-EOB-DTPA-enhanced MRI. Therefore, Gd-EOB-DTPA-enhanced MRI has the potential to serve as an imaging biomarker for identifying subpopulations of patients who are unlikely to respond well to ICIs therapy and can be excluded from clinical trials or real-world practice involving ICIs treatment. As reported, a newly identified subclass of HCC exhibits distinctive characteristics of strong activation of interferon signaling and immune activation along with the presence of CTNNB1 mutations; it is probably an inflammatory tumor [3]. Kubo et al. [56] reported that patients who had HCC with iso-high intensity in the HBP of Gd-EOB-DTPA-enhanced MRI exhibit large proportion of CTNNB-1 mutation; this also suggests the potential value of Gd-EOB-DTPA-enhanced MRI in evaluating HCC immunotherapy.

**Table 1:** Existing papers that investigated the use of magnetic resonance imaging to predict the efficacy of immunotherapy in hepatocellular carcinoma.

Title	Authors	Journal	Date	Conclusions
Evaluating the Role of Hepatobiliary Phase of Gadoxetic Acid-Enhanced Magnetic Resonance Imaging in Predicting Treatment Impact of Lenvatinib and Atezolizumab plus Bevacizumab on Unresectable Hepatocellular Carcinoma	Sasaki R, Nagata K, Fukushima M et al. [49]	*Cancers* (Basel)	2022	The hepatobiliary phase of Gd-EOB-DTPA-enhanced MRI was useful for predicting the therapeutic effect of atezolizumab plus bevacizumab therapy on unresectable HCC
Characteristics and Lenvatinib Treatment Response of Unresectable Hepatocellular Carcinoma with Iso-High Intensity in the Hepatobiliary Phase of EOB-MRI	Kubo A, Suda G, Kimura M et al. [56]	*Cancers* (Basel)	2021	The response to lenvatinib does not differ between HCC with and without iso-high intensity in the hepatobiliary phase of Gd-EOB-DTPA-enhanced MRI. CTNNB-1 mutations are association with iso-high intensity in the hepatobiliary phase of Gd-EOB-DTPA-enhanced MRI.
Higher Enhancement Intrahepatic Nodules on the Hepatobiliary Phase of Gd-EOB-DTPA-Enhanced MRI as a Poor Responsive Marker of Anti-PD-1/PD-L1 Monotherapy for Unresectable Hepatocellular Carcinoma	Aoki T, Nishida N, Ueshima K et al. [50]	*Liver Cancer*	2021	The intensity of the nodule on the hepatobiliary phase of Gd-EOB-DTPA-enhanced MRI is a promising imaging biomarker for predicting unfavorable response with anti-PD-1/PD-L1 monotherapy in patients with HCC
Gd-EOB-DTPA-MRI Could Predict WNT/β-Catenin Mutation and Resistance to Immune Checkpoint Inhibitor Therapy in Hepatocellular Carcinoma	Kudo M. [48]	*Liver Cancer*	2020	The signal intensity on HBP of Gd-EOB-DTPA-enhanced MRI can be used to noninvasively predict the effect of ICI monotherapy

## 6. Prospects of MRI in the Era of Hepatocellular Carcinoma Immunotherapy

Recently, multi-omics approaches have been used to elucidate the underlying mechanisms of immunotherapy response in HCC through the characterization of the tumor immune microenvironment features. Research has showcased the utilization of multi-omics signatures, specifically matrix metalloproteinase (MMP9), as a means to identify patients with distinct prognoses and varying responses to immunotherapy in HCC [57]. This study holds the potential to unravel the immune status of HCC and offer valuable indicators for predicting the response to immune checkpoint therapy. Additionally, a multi-omics study showed that palmitic acid-induced lipid accumulation in HCC cells upregulated PD-L1 expression and promoted immunosuppressive phenotypes of cocultured macrophages and fibroblasts. Notably, patients with steatotic HCC, as confirmed via chemical-shift MR imaging, exhibited significantly prolonged progression-free survival (PFS) upon receiving a combined immunotherapy regimen involving anti-PD-L1 and anti-VEGF antibodies [58]. Moreover, a tumor immune barrier (TIB) structure was identified via combined spatial transcriptomics with single-cell RNA-sequencing and multiplexed immunofluorescence that determine the efficacy of immunotherapy in patients with HCC receiving anti-PD-1 treatment [59]. However, so far, there is a lack of effective imaging methods to conveniently and non-invasively predict these multi-omics features.

Our analysis suggests that with the advancement of precision medicine, it is inadequate to simply employ radiomics and machine learning techniques for assessing the tumor immune microenvironment and predicting the efficacy of immunotherapy. Instead, it is crucial to explore stable radiomic features in these multicenter, large-scale, standardized HCC MRI datasets, and combine them with genomic, proteomic, and other pathological features to elucidate the biological mechanisms underlying these radiomic characteristics.

Recently, artificial intelligence (AI) has emerged as a unique opportunity to improve the full spectrum of HCC clinical care, including HCC risk prediction, diagnosis, and prognostication [60]. Additionally, its powerful computational capabilities provide it with significant advantages for integrating multi-omics data to detect HCC. The progress in molecular measurement technologies have facilitated the profiling of various molecular features of HCC (such as genome, transcriptome, proteome, and metabolome) at multiple levels. Making these datasets publicly available not only strengthens the capacity to extract valuable information from past studies but also offers the opportunity to utilize them for comprehending HCC mechanisms, developing new therapeutics, and identifying potential biomarkers for treatment response. The emerging AI techniques can be harnessed to discover novel targets and drugs, and to provide guidance for enhanced HCC treatment decisions [61]. The future use of AI and multi-omics in clinical workflow is detailed in Figure 1.

However, AI approaches, which commonly involve intricate layers of mathematical computations, pose significant challenges in gaining a comprehensive understanding of how data are transformed throughout the entire network. The pursuit of explainable AI is currently a growing area of research, with the primary objective of unveiling the inner workings of neural networks. Several research directions are being explored to demystify the ‘black boxes’ of neural networks. Key approaches include enhancing the transparency of networks, unraveling the semantics of different components, and generating post hoc explanations [62].

**Figure 3:**
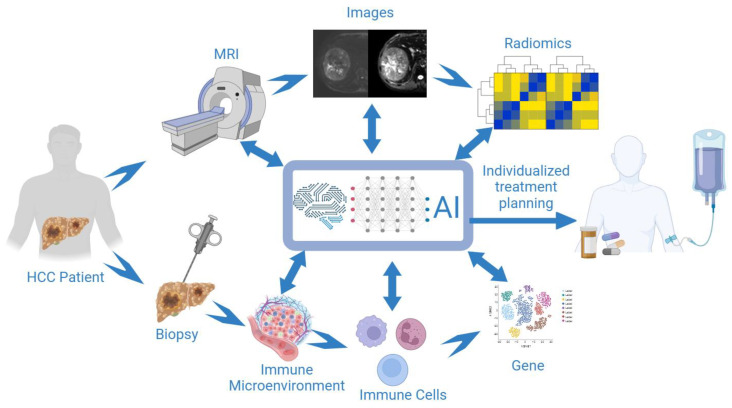
The future use of artificial intelligence (AI) and multi-omics in clinical workflow. Combined multi-parameter MRI with multi-omics and AI techniques to develop an effective tool for identifying patients who would benefit from immunotherapy and predicting the tumor response to immunotherapy (figure created with BioRender.com).

Ensuring the interpretability of radiomics features and AI decisions is a shared concern across all current and future AI applications. Enabling various features of radiomics to have biological interpretability has always been a matter of great concern and a significant future research direction for radiologists. The notion of interpretability encompasses a specific array of techniques that empower users to grasp the inner workings and decision-making process of radiomics or AI models. It provides valuable insights into the essential factors influencing predictions and facilitates the understanding of potential biases. This transparency plays a crucial role in establishing the necessary trust to encourage medical professionals to embrace computer-aided devices that may be employed in the future (Figure 3).

## 7. Conclusions

Multiple potential biomarkers for predicting ICI response of HCC have been explored and applied clinically. Yet there is no robust evidence to prove their clinical value in predicting immunotherapeutic response for patients with HCC. The identification of predictors which respond to ICIs is an urgent need and major challenge. Further studies could combine multi-parameter MRI with various AI techniques to develop and validate an effective tool for identifying the patients who would benefit from immunotherapy, and which can be used for predicting the tumor response to immunotherapy.
